
JOURNAL INFORMATION
==============================
NLM Title Abbreviation: Behav Neurol
Journal ID: Behav Neurol
NLM Journal ID: 8914585
Journal ID: BN

Behavioural Neurology

ISSN: 0953-4180
EISSN: 1875-8584
Publisher: Wiley

ARTICLE INFORMATION
==============================
PMCID: PMC5294265
PMID: 22207417
DOI: 10.3233/BEN-2012-0343
Article ID: 901791
Article version: 1
Subjects: Introduction

Topics in Dementia: Papers from the WFN-RGACD 2010 Meeting

Manes Facundo 1 2 3 *
1 Institute of Cognitive Neurology (INECO) Buenos Aires Argentina
2 Laboratory of Cognitive Neuroscience, Universidad Diego Portales, Santiago Chile
3 Institute of Neurosciences, Favaloro University, Buenos Aires Argentina
*Facundo Manes: fmanes@ineco.org.ar
Print publication date: 2012
Electronic publication date: 2011 Dec 29
Volume: 25
Issue: 1
First page: 1
Last page: 2
Received 2011 Dec 26; Accepted 2011 Dec 26
Copyright: Copyright © 2012 Hindawi Publishing Corporation and the authors.
Copyright year: 2012
License: This is an open access article distributed under the Creative Commons Attribution License, which permits unrestricted use, distribution, and reproduction in any medium, provided the original work is properly cited.
License URL: https://creativecommons.org/licenses/by/3.0/

==============================
Behavioural Neurology 25 (2012) 1–2
DOI 10.3233/BEN-2012-0343
IOS Press

Introduction

### Topics in Dementia: Papers from the WFN-RGACD 2010 Meeting

Facundo Manes
*Institute of Cognitive Neurology (INECO), Buenos Aires, Argentina*
*Laboratory of Cognitive Neuroscience, Universidad Diego Portales, Santiago, Chile*
*Institute of Neurosciences, Favaloro University, Buenos Aires, Argentina*
*E-mail: fmanes@ineco.org.ar*

On June 15–18, 2010, the World Federation of Neurology's Research Group on Aphasia and Cognitive Disorders (WFN-RGACD) held its biannual meeting in Istanbul, Turkey. Research presented during the meeting covered a wide array of topics in the field of behavioral neurology and represented a clear reflection of creativity and innovative research aimed at deepening our understanding of some of the most fundamental aspects of this field. A large portion of the ongoing investigations presented during the meeting focused on different types of dementia. This special issue of *Behavioural Neurology* surveys an interesting range of topics in the field of dementia. As a whole, the collection of papers included in this special issue represents some of the trends in dementia research which have been most fruitful in generating innovative findings with direct implications for assessment, diagnosis, and treatment of different patient populations with dementia.

Two articles included in this special issue demonstrate the relevance of incorporating some specific tools in the study of dementia. Görsev Yener and collaborators employed auditory delta evoked- and event-related oscillations obtained from EEG data to compare patients with Alzheimer disease with healthy controls. Their article demonstrates how electrophysiological data can be exploited to incorporate objective measures that may compliment behavioral and cognitive assessment of patients with dementia. Andreas Hinz applies mathematical modeling centered on graph theory to dissect and further analyze performance on tasks that have been extensively used in dementia: the tower tasks (e.g. Tower of London and Towe of Hanoi). The article provides detail on the mathematical reasoning behind this application of math to cognitive assessment, and offers conclusions as to why graph theory can bring a better understanding neuropsychological performance and could thus help develop new puzzle tests for cognitive assessment.

Four articles then explore particular aspects of cognitive functioning in different groups of dementia. Faye Corbette and collaborators offer a novel perspective on the semantic impairment of patients with Alzheimer disease. Their article explores the qualitative changes in semantic performance as disease severity progresses through time. Julie Snowden and her collaborators examine the performance of patients with semantic dementia with predominantly right and predominantly left temporal lobe atrophy on famous face, famous name, and general semantic tasks. By doing so, they report findings that challenge previously conceived semantic models which have been very influential in the field. Ezequiel Gleichgerrcht and collaborators have investigated the decision making profile of patients with primary progressive aphasia, and compared their performance to that of patients with behavioral variant frontotemporal dementia and healthy controls. Their findings in the context of performance in language tasks offer insight into the decision making profile of a type of dementia whose behavioral disturbances are yet to be comprehensively characterized. Finally, Laurie Miller and collaborators provide evidence of differences in the cognitive mechanisms underlying facial emotion pro-

ISSN 0953-4180/12/$27.50 © 2012 – IOS Press and the authors. All rights reserved cessing impairment in different populations of patients with dementia, including behavioral variant frontotemporal dementia, semantic dementia, and Alzheimer disease. Their assessment through three different facial emotion processing tasks furthers our understanding about the multifactorial impairment of facial emotion processing within each pathological group.

The quality of research presented in this special issue represents merely a portion of the creative and stimulating intellectual exchange of researchers from around the world who participated in the 2010 meeting of the WFN-RGACD.
